# The use of self-management strategies for problem gambling: a scoping review

**DOI:** 10.1186/s12889-019-6755-8

**Published:** 2019-04-29

**Authors:** Flora I. Matheson, Sarah Hamilton-Wright, David T. Kryszajtys, Jessica L. Wiese, Lauren Cadel, Carolyn Ziegler, Stephen W. Hwang, Sara J. T. Guilcher

**Affiliations:** 1 MAP Centre for Urban Health Solutions, St. Michael’s Hospital, 30 Bond Street, Toronto, ON M5B 1W8 Canada; 2 Health Information Sciences Library, University of Toronto, Toronto, ON Canada; 3 Leslie Dan Faculty of Pharmacy, University of Toronto, Toronto, ON Canada

**Keywords:** Self-management, Gambling, Problem gambling, Self-efficacy, Self-help, Coping, Strategies, Coping skills, Self-exclusion, Gamblers, Scoping review

## Abstract

**Background:**

Problem gambling (PG) is a serious public health concern that disproportionately affects people experiencing poverty, homelessness, and multimorbidity including mental health and substance use concerns. Little research has focused on self-help and self-management in gambling recovery, despite evidence that a substantial number of people do not seek formal treatment. This study explored the literature on PG self-management strategies. Self-management was defined as the capacity to manage symptoms, the intervention, health consequences and altered lifestyle that accompanies a chronic health concern.

**Methods:**

We searched 10 databases to identity interdisciplinary articles from the social sciences, allied health professions, nursing and psychology, between 2000 and June 28, 2017. We reviewed records for eligibility and extracted data from relevant articles. Studies were included in the review if they examined PG self-management strategies used by adults (18+) in at least a subset of the sample, and in which PG was confirmed using a validated diagnostic or screening tool.

**Results:**

We conducted a scoping review of studies from 2000 to 2017, identifying 31 articles that met the criteria for full text review from a search strategy that yielded 2662 potential articles. The majority of studies examined self-exclusion (39%), followed by use of workbooks (35%), and money or time limiting strategies (17%). The remaining 8% focused on cognitive, behavioural and coping strategies, stress management, and mindfulness.

**Conclusions:**

Given that a minority of people with gambling concerns seek treatment, that stigma is an enormous barrier to care, and that PG services are scarce and most do not address multimorbidity, it is important to examine the personal self-management of gambling as an alternative to formalized treatment.

**Electronic supplementary material:**

The online version of this article (10.1186/s12889-019-6755-8) contains supplementary material, which is available to authorized users.

## Background

Problem gambling (PG) is a serious chronic health condition and public health concern that affects between 0.12 and 5.8% of the general population worldwide [1], and up to 7% in some studies [2]. Those who are most susceptible to PG often experience other complex health and social concerns such as homelessness, mental health issues, substance use disorders, and incarceration [3–5]. Existing services are often not integrated and thus are not designed to address concurrent concerns with PG [6]. Among treatment seeking individuals, there is a need to increase awareness of existing PG-related services and supports [6]. Further, while there are PG interventions that have demonstrated effectiveness, they can be inaccessible to many vulnerable groups due to barriers such as geographical distance, long waitlists, and treatment costs [6–12]. Barriers to treatment of PG are reflected in low rates of treatment-seeking, as some research has found that only 1 in 10 people experiencing PG seek treatment compared to 1 in 5 people with alcohol-related disorders [13, 14]. There are also important individual barriers to help-seeking that must be considered. Factors such as problem denial, a fear of stigmatization, the belief in the normalization of gambling, and the belief that gambling is not a disease are cited as reasons why many do not seek formal treatment [12, 15–18]. Several qualitative studies found that emotions such as pride and shame discourage help-seeking [19–21]. In particular, some individuals felt a sense of shame in admitting their problem in a group context, and feared additional stigmatization when disclosing their struggles to strangers [17]. Overcoming individual barriers related to cognitions and beliefs about gambling is necessary before a person can meaningfully commence help-seeking behaviour, whether it be seeking formal treatment services or making the decision to engage in self-management. Increased public education and awareness of PG symptomology and treatment plays an important role in reducing shame, stigma and denial in individuals [18]. Likewise, self-management may be an attractive alternative to formalized treatment for individuals concerned with shame and the perceptions of others.

To help address these barriers, self-management may be used as an adjunct or as an alternative to treatment, and may also be effective for people experiencing complex needs such as those with PG [22]. Self-management is defined as, “an individual’s ability to manage the symptoms, treatment, physical and psychosocial consequences and lifestyle changes inherent in living with a chronic condition. Efficacious self-management encompasses the ability to monitor one’s condition and to affect the cognitive, behavioural and emotional responses necessary to maintain a satisfactory quality of life” (p. 178) [23]. Self-management interventions are rooted in improving an individual’s self-efficacy to manage symptoms through mastery of skills such as problem solving, decision making, resource utilization, forming a patient/health care provider partnership [24, 25]), modeling, interpreting physical symptoms, and social persuasion [25]. Self-management interventions can be provided individually or in a group setting, and can be facilitated by technology in either context [26, 27].

There is extensive empirical evidence for self-management strategies for a range of chronic health issues [22]. Some preliminary research suggests that self-management interventions may be useful for treating addictions to alcohol [28–30] and cannabis [31]. A review found that self-administered treatments (e.g., self-help book) are effective for treating mild alcohol abuse while more severe cases show better outcomes with the use of therapist mediated treatments [32]. Self-management treatments have been used to manage behavioural issues such as nail-biting, poor physical activity, poor diet and excessive internet use [33]. Whiteman et al. [34] conducted a meta-analysis of programs that teach self-management training (e.g., interpersonal skills, trigger identification) and found that the training is effective for dealing with co-occurring mental and physical health issues such as bipolar disorder and asthma. Despite the effectiveness, there is some evidence that self-help treatments may not be well-suited for individuals experiencing severe psychological problems (e.g., personality disorders) and significant interpersonal difficulties [32]. In these more severe cases where individuals may lack capacity, clinician-administered treatments may be bettered suited.

Despite the promising evidence that self-management strategies can be effective for persons with chronic health concerns and complex needs, reviews exploring the current state of the literature on a wide range of self-management strategies for PG are limited. For instance, Raylu et al. [35] reviewed self-help treatment studies up to the year 2008 and found that research on these treatments for PG was still in its infancy. The researchers noted that most studies focused on only two strategies (i.e., self-help manuals and audiotapes), and discussed the importance of exploring a wider range of self-help strategies. In line with this work, Rodda et al. [36] identified six change strategies described by online counselling session clients, and in later examined the perceived helpfulness of 15 cognitive change strategies, noting differences in the helpfulness of particular strategies based on gambling severity as well as age [37]. Although the authors provide an extensive list of strategies, they acknowledge that some strategies may have been missed or conceptualized differently than in past literature. The objective of this scoping review was to build on this work and identify and describe what was reported in the literature on PG self-management strategies.

## Methods

### Criteria for inclusion and exclusion

The basis for the methodology of this scoping review is the five-stage approach suggested by Arksey and O’Malley: (1) identify the research question, (2) identify relevant studies, (3) select relevant studies, (4) chart the data, (5) collate, summarize and report the results [38]. We followed the guidelines of the PRISMA-P [39] as the PRISMA-ScR [40] was not available during the review process. We completed the PRISMA-ScR as a Additional file 1 document to this paper. We did not provide a critical assessment of the quality of the evidence as this is a developing area of research.

Studies were included if the authors examined PG self-management strategies used by adults (18+) in at least a subset of the sample; and PG was confirmed using a validated diagnostic or screening tool. We defined self-management strategies as techniques used to self-manage gambling activities independently of clinician support. The independent use of a strategy includes use after a therapy session, use after being introduced by a researcher, and use outside of interactions with researchers and therapists. Some examples of self-management strategies that fit this definition include money limiting strategies, self-management components of Cognitive Behavioural Therapy (CBT; e.g., workbooks, thought records, journaling), coping strategies, and mindfulness. We included randomized controlled trials, observational (cohort, cross-sectional, case-control), descriptive, qualitative, and mixed methods studies. We examined systematic, scoping, realist, and narrative reviews to identify additional studies that met our inclusion criteria. Studies were excluded if they only included face-to-face treatment without a self-management component, peer support groups such as Gambler’s Anonymous or online discussion forums, strategies that focused only on gaining knowledge and awareness, and studies examining treatment-seeking behaviour. We also excluded non-peer reviewed works such as reports, theses, dissertations, conference presentations, conference papers, books, book reviews, case studies, trial papers and protocols.

### Search strategy

We collected studies for our review using a search strategy developed by an information specialist and the project team (see Additional file 2 for the full MEDLINE strategy). The following databases were searched in June 2017: Medline, PsycINFO, Embase, the Cochrane Library, CINAHL, Applied Social Sciences Index & Abstracts, International Bibliography of the Social Sciences, ProQuest Dissertations & Theses Global, Social Services Abstracts, and Sociological Abstracts.

Our selection of databases ensured interdisciplinary coverage of research in social sciences, allied health professions, nursing and psychology. We used search terms that included a combination of keywords and subject headings for the concepts of gambling and self-management, combined with the Boolean operator “AND.” We limited the search to articles published in English or French between 2000 and June 28, 2017. Papers published in French with an English equivalent translation were considered for the review, but none were identified. We supplemented the database searches with cited reference searching. Citations were managed using EndNote.

### Study selection

**Fig. 1:**
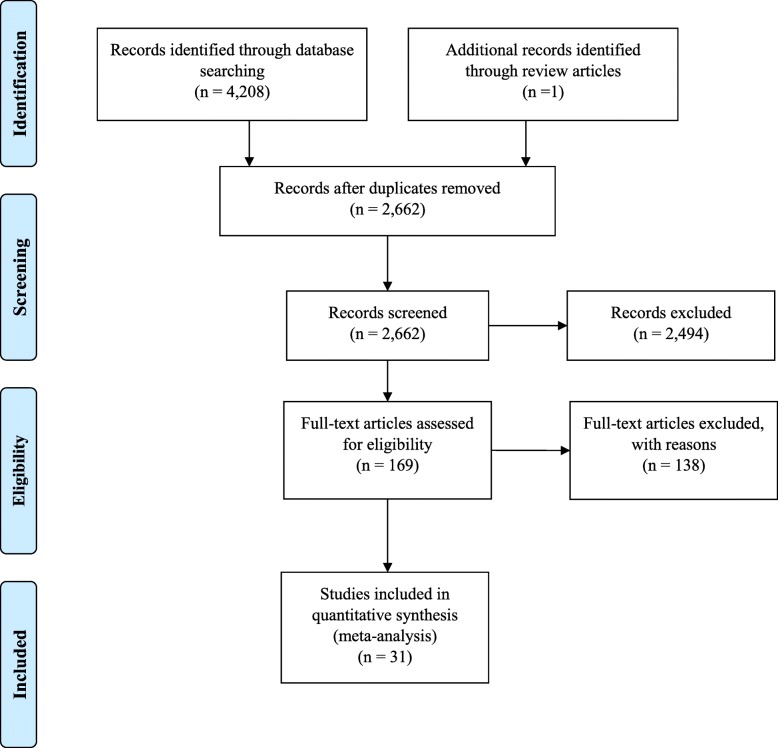
Flow diagram of study selection Table 1 Characteristics of studies included the review

The next step in the review was to select relevant studies. First, three team members independently reviewed 30 studies to pilot the eligibility criteria for the title and abstract review. Any conflicts were resolved through a larger team discussion. The team refined the inclusion and exclusion criteria based on the pilot and then independently reviewed titles and abstracts of all 2662 studies identified through the search strategy. A total of 169 studies were identified as eligible for full text review. Three team members piloted 17 studies for the full-text review, and then independently reviewed the 169 studies for eligibility and extracted data from 31 articles that met the inclusion criteria. See Fig. 1 for the flowchart of study selection and screening.

### Data extraction

To chart our data, three team members independently extracted information from eligible publications using a data extraction tool the team developed, piloted and modified. The tool provided detailed instructions and formatting guidelines for the data extraction and charting. The following information was extracted using the tool: publication details (authors, publishing year, journal), research objectives, type and description of self-management strategy, methodology, method, outcome measures, sample information and demographics, information on tools used to measure PG, qualitative findings, and authors’ main conclusions. For the purposes of this review, self-management strategies were organized into four categories: behavioural self-management (*n* = 19), cognitive self-management (*n* = 2), coping skills/styles (*n* = 12), and multi-part interventions (*n* = 16).

## Results

### Description of studies

Table 1 describes the characteristics of included studies. Most studies were conducted in Canada (*n* = 11), Australia (*n* = 7), and the United States (*n* = 5). Studies were also conducted in Finland, Sweden, Germany, Switzerland, Spain, Greece, Singapore, and New Zealand. The majority of studies included were quantitative (*n* = 24), with fewer being qualitative (*n* = 3) or mixed-methods (*n* = 4). Most studies had a mix of males and females within their samples (*n* = 27); only two studies used an all-male sample and two studies used an all-female sample. The majority of studies (*n* = 17) did not report on race or ethnicity. Of the studies that reported race, most had a majority white sample (n = 11). Some studies reported on ethnicities rather than race (*n* = 6), with samples in which the majority of participants were Canadian (n = 4), Australian (n = 1), and Chinese (n = 1). Three studies were conducted in a clinical setting. One study included participants from a rural setting, and one study included participants with low-income backgrounds. Of the 31 studies, 16 included participants with mental health and/or substance use comorbidities. Health comorbidities with PG included mood disorders (depression, manic depression, bipolar disorder, dysthymia, suicide ideation), substance use disorders (alcoholism, drugs), impulse control disorders (compulsive buying, compulsive sexual behaviour, kleptomania), anxiety disorders (social phobia, obsessive-compulsive disorder, panic disorder), eating disorders, and experiences of emotional abuse, sexual abuse, physical abuse, loss, stress, and head injury.

**Table 1:** Characteristics of studies included the review

Authors; Country	Method	N, sex	Age in years M (SD), Range^a^	Race/ Ethnicity	Clinical Setting (Y/N)^b^	Special Population^c^	Health Comorbidities with Problem Gambling
Avery et al. (2008) [41]; Not reported	Mixed	*N* = 136; 100% female	18–24 (2.2%); 25–34 (15.4%); 35–44 (28.7%); 45–54 (35.3%); 55–64 (11.8%); 65+ (3.7%); Missing (2.9%)	White (91.9%), African-American (2.9%), Asian American (2.2%), Hispanic (0.7%), Other (1.5%), Missing (0.7%)	No	No	Mental illness (depression, bipolar disorder and dysthymia), drug addiction, alcoholism
Boughton et al. (2016) [42]; Canada	Mixed	*N* = 25; 100% female	56.0	White (85%)	No	No	Emotional abuse, sexual abuse, physical abuse, experiences of loss, trauma, mental health issues (depression, anxiety, panic, manic depression, anger), suicide ideation
Campos et al. (2016) [43]; United States^d^	Quant.	*N* = 87; 76% male, 24% female	43.4 (10.8); 45.1 (11.0)	White (44%), African American (32%), Asian (12%), Other (12%)	No	Low income	Generalized anxiety disorder, major depressive disorder, dysthymia, psychotic disorders, alcohol and/ or marijuana abuse/ dependence
Casey et al. (2017) [44]; Australia^e^	Quant.	*N* = 174; 41% male, 59% female	44.8 (9.0); 44.1 (10.5); 44.2 (9.5)	White (81%), Asian (2%), Other (17%); White (76%), Asian (6%), Other (18%); White (82%), Asian (2%), Other (16%)	No	No	Depression, anxiety and stress, alcohol use
Castren et al. (2013) [45]; Finland	Quant.	*N* = 471; 69% male, 31% females	34.5 (11.8)	Not reported	Yes	No	Alcohol consumption, depression
Cunningham et al. (2012) [46]; Canada	Quant.	N = 176^f^, 51% male, 49% female	47.6 (13.4)	Not reported	No	No	Not reported
Forsstrom et al. (2017) [47]; Sweden	Qual.	*N* = 20; 95% male, 5% female	42.15 (12.70)	Not reported	No	No	Not reported
Grant et al. (2011) [48]; United States^g^	Quant.	*N* = 68 (35 follow-up); 62% male, 38% female	49.55; 48.51	White (94%)	No	No	Mood disorder (major depressive disorder, dysthymia, depressive disorder not otherwise specified), impulse control disorder (compulsive buying, compulsive sexual behaviour, or kleptomania), anxiety disorder (social phobia, obsessive-compulsive disorder, panic disorder), eating disorder
Hayer and Meyer (2011) [49]; Australia, Germany, Switzerland	Quant.	*N* = 152; 72% male; 28% female	41.3	Not reported	No	No	Not reported
Hing, Russell, Gainsbury et al. (2015) [50]; Australia	Quant.	*N* = 620; 80% male, 20% female	37.6 (13.1)	Not reported	No	No	Psychological distress
Hing, Sproston et al. (2017) [51]; Australia	Quant.	*N* = 860; 68% male, 32% female	49.5 (15.9)	Not reported	No	No	Not reported
Hing, Cherney, et al. (2015) [52]; Australia	Qual.	*N* = 25; 100% male	39.9 (14.1)	Not reported	No	No	Not reported
Hing, Russell, Tolchard, et al. (2015) [53]; Australia	Quant.	*N* = 103; 56% male, 44% female	66.2 (6.5)	Not reported	No	No	Alcoholism
Hodgins (2005) [54]; Canada	Quant.	*N* = 102; 48% male, 52% female	46 (9)	Canadian (89%), Native (2%), Other (7%)	No	No	Not reported
Hodgins et al. (2009) [55]; Canada	Quant.	*N* = 314; 45% male, 55% female	Not reported	Not reported	No	No	Mental health disorders, alcohol abuse or dependence, major depressive disorder, bipolar disorder (lifetime)
Hodgins et al. (2001) [56]; Canada	Quant.	N = 102; 48% male, 52% female	46 (9)	Canadian (89%), Native (2%), Other (7%)	No	No	Not reported
Hodgins et al. (2007) [57]; Canada	Quant.	*N* = 169; 58% male, 42% female	32 (11.2)	Canadian (89%), Other (7%), Native/ Metis (4%)	No	No	Depression, alcohol or drug addiction, emotional/ mental health difficulties
Hodgins, Currie, el-Guebaly, Peden et al. (2004) [58]; Canada^h^	Quant.	N = 102 (52 follow-up); 44% male, 56% female	46.0 (10.0)	English Canadian (90%), Aboriginal (2%), French Canadian (2%), Other (6%)	No	No	Not reported
Jauregui et al. (2017) [59]; Spain^i^	Quant.	*N* = 274; 100% male	39.3 (11.8); 33.4 (11.9)	Not reported	Yes	No	Depression, anxiety
Labrie et al. (2012) [60]; United States^j^	Quant.	*N* = 248; 52% male, 48% female; 66% male, 34% female	44.0 (14.0); 49.0 (10.0)	White (74%), Hispanic (15%); White (66%), Hispanic (3%)	No	Rural	Mental/ emotional problems, drug/ alcohol problems
Ladouceur et al. (2000) [61]; Canada	Quant.	*N* = 220; 62% male, 38% female	41.0	Not reported	No	No	Not reported
Ladouceur et al. (2007) [62]; Canada	Quant.	*N* = 161; 60% males; 40% females	43.5 (12.3)	Not reported	No	No	Not reported
Lalande and Ladouceur (2011) [63]; Canada^k^	Mixed	N = 20; *N* = 65; 67% male, 33% female; 66% male, 34% female	47.7 (13.4); 43.6 (15.2)	Not reported	No	Low-income	Not reported
Linardatou et al. (2014) [64]; Greece^l^	Mixed	*N* = 45; 95.5% male, 4.5% female; 90% male; 10% female	42.6	Not reported	No	No	Depression, anxiety, stress
Luquiens et al. (2016) [65]; Not reported	Quant.	*N* = 1122; 92% male; 8% female	34.7	Not reported	No	No	Not reported
Martin (2013) [66]; United States	Quant.	*N* = 60; 60% male; 40% female	Underclassmen (Freshman, Sophomore) (88.3%); Upperclassmen (Junior/ Senior) (11.7%)	Caucasian (non-Hispanic) (71.7%), African American (20%); Hispanic or Latino (5%), American Indian or Alaskan Native (1.7%), Other (1.7%)	No	No	Not reported
Moore et al. (2012) [67]; Australia	Quant.	*N* = 303; 39% male, 61% female	26.4 (10.1)	Australian (79%), European (14%), Asian (7%)	No	No	Not reported
Nelson et al. (2010) [68]; United States	Quant.	*N* = 113; 45% male, 55% female	45.0 (10.0)	White (80.5%), Black or African-American (16.8%), Other (2.7%), Hispanic or Latino (0.9%)	No	No	Substance use, mental health problems
Subramaniam et al. (2017) [69]; Singapore	Qual.	N = 25; 72% male, 28% females	66.2 (6.5)	Chinese (64%), Indian (16%), Other (12%), Malay (8%)	No	Adults (60+)	Not reported
Toneatto et al. (2014) [70]; Canada^m^	Quant.	*N* = 18; 44.4% male, 55.6% female; 66.7% male, 33.3% female	41.7 (11.0); 46.6 (11.8)	Not reported	No	No	Current emotional distress
Townshend (2007) [71]; New Zealand	Quant.	*N* = 35; 60% male; 40% female	18–73	Not reported	Yes	No	Head injury, substance use disorder, other mental health disorder

^a^Values reported when available

^b^Clinical setting includes participants (full or subset of sample) recruited from treatment centers, in-patient programs, residential treatments or therapist-facilitated out-patient treatment programs

^c^Special designation includes participants (full or subset of sample) who belong to populations such as veteran, homeless/in poverty, correctional or elderly

^d^Age information provided by condition (workbook only; workbook plus therapist guidance)

^e^Age and Race/ Ethnicity information provided by condition (internet-based CBT, internet-based MFS, waitlist, respectively)

^f^Completed at least one follow-up

^g^Age information provided by condition (imaginal desensitization; Gambler’s Anonymous control)

^h^Same sample as Hodgins et al. (2001)

^i^Age and Race/ Ethnicity information provided by condition (pathological gambling group; non-pathological gambling group)

^j^Sex, Age and Race/ Ethnicity information provided by sample (Nevada, US; Massachusetts, US)

^k^Ns presented by study (Pilot; Main Study); Sex and Age provided by sample (problem gambling subset; non-problem gambling subset)

^l^Sex presented by condition (intervention; control)

^m^Sex, Age information presented by condition (Mindfulness plus CBT; waitlist)

### Self-management strategies

Table 2 provides detailed descriptions and key findings regarding the self-management strategies. From a total of 31 studies, we identified 24 self-management strategies. Most studies examined one strategy (*n* = 25), three included two strategies [41, 64, 70], three included three or more strategies [59, 67, 69].

**Table 2:** Self-management strategies described in the included studies

Authors	Self-Management Strategy	Details/ Description of Self-Management Strategy	Key Findings and Implementation Considerations related to the Self-Management Strategy
Avery et al. (2008) [41]	• Alternative activity scheduling • Self-exclusion	Alternative Activity Scheduling • Person plans non-gambling activities to take the place of gambling (e.g., joining social groups, making plans to fill time)	• Gender differences identified for motivation to stop gambling and supports sought; most women used GA, professional help (or both) • A small proportion (10%) of women recovered on their own from PG using alternative activity scheduling and self-exclusion and had lower PG scores than those receiving professional or GA help
		Self-exclusion • Person relocates away or self-bans from gambling establishments for a fixed period of time.	
Boughton et al. (2016) [42]	Workbook (web-based)	• Workbook contains 12 weekly modules • Modules cover: change process, urges and relapses, exploration of how thoughts impact feelings, mindfulness, stress management, relationships, emotional regulation, goals.	• The workbook was well received by participants • Web- and phone-based group sessions are effective to expand PG services
Campos et al. (2016) [43]	Workbook	• Person reads and completes exercises in workbook on topics such as awareness of PG behaviour and reasons for gambling, motivation for behaviour change, money limiting, changing faulty cognitions about gambling, tools to maintain abstinence.	• Use of self-help workbooks helps reduce PG symptoms and money spent gambling, but using a workbook with therapist guidance had superior outcomes
Casey et al. (2017) [44]	Internet-based cognitive behavioural therapy	• Six weeks of CBT sessions with exercises. • Sessions cover: awareness of triggers and strategies to cope with urges, challenging thinking errors and replacing them with helpful thoughts, debt management, imaginal desensitization, relaxation training, problem solving, goal setting, emotions, and maintenance and relapse prevention.	• CBT was associated with reduced gambling severity, other beneficial PG and mental health outcomes, and greater satisfaction after initial treatment and 12-month follow-up • Online treatments for gambling may be a valuable tool in increasing help-seeking and treatment engagement
Castren et al. (2013) [45]	Internet-based cognitive behavioural therapy	• 8 weeks of CBT program with weekly module. • Each CBT module contains information, exercises and homework covering: Psychoeducation and motivation, recognizing high risk situations and triggers, identifying social consequences of gambling, recognizing erroneous thoughts, safe ways to manage high-risk situations, money management.	• CBT was associated with reduced gambling-related problems, urges, impaired control of gambling, alcohol consumption, social consequences, gambling-related cognitive erroneous thoughts and depression
Cunningham et al. (2012) [46]	Personalized feedback tool	• Person completes Problem Gambling Severity Index (PGSI) and Gambling Cognitions Questionnaire. • Feedback tool provides a summary of the individual’s PGSI scores and summary of cognitive distortions that they endorsed (along with summary of the error of each of these beliefs); a list of techniques to lower gambling risks; a comparison of the amount of money the individual spent in the past year with the average amount of money spent by Canadians of the same sex.	• No evidence for the impact of normative personalized feedback; however, participants who received partial feedback (without norms) reduced the number of days gambled compared to those not receiving the intervention • Personalized feedback interventions were well received and Internet-based personalized feedback tools may improve access to interventions
Forsstrom et al. (2017) [47]	Personalized feedback tool	• Individual completes a weekly risk assessment test. • Feedback tool provides a risk level based on comparison of gambling patterns with other users, a detailed history of gambling habits and advice about how to limit time and money spent on gambling.	• Participants had a positive view of the tool’s content, which should have promoted use; however, repeated use was low • A lack of feedback from the tool and confusion when signing up may have affected usage; offering users direct feedback may increase usage
Grant et al. (2011) [48]	Imaginal Desensitization	• 6 sessions of imaginal desensitization plus motivational interviewing (IDMI) over an 8-week period • Audio-recordings of three gambling scenarios are played three times per day which prompt the user to use relaxation coping strategies to cope with urges the scenarios elicit.	• The intervention reduced PG urges and behaviour; effects were largely maintained for 6 months • Participants cited their audiotapes as the primary reason for their improvement.
Hayer and Meyer (2011) [49]	Self-exclusion	• Once registered, the individual is banned from gambling venues and websites for a fixed period of time.	• Mostly men and middle-aged individuals place themselves on exclusion lists; top motives to self-exclude involve financial difficulties • Self-exclusion is effective when used in combination with additional counseling
Hing, Russell, Gainsbury et al. (2015) [50]	Self-exclusion	• Once registered, the individual is banned from gambling venues and websites for a fixed period of time.	• Land-based gamblers are more likely to use self-exclusion strategies than problem Internet gamblers
Hing, Sproston et al. (2017) [51]	Responsible Gambling Strategies • Money and time limiting	• When gambling, Person carries limited money, stops gambling once it is spent, does not carry bank card, does not re-gamble any wins, and places smaller bets.	• Setting money limits and balancing gambling with other activities predict non-harmful gambling
Hing, Cherney, et al. (2015) [52]	Self-exclusion (online)	• Individual blocks themselves from specific gambling websites using website blocking software.	• Limiting strategies had variable success; most felt that operators needed to implement more responsible gambling measures (e.g., removal of credit betting, imposed bet limits, gambling help pop-up messages, restricted promotions/ advertising)
	Money limit setting (online)	• Individual deposits monetary amounts to bet at the outset of gambling episode and they prohibit themselves from credit betting.	
Hing, Russell, Tolchard, et al. (2015) [53]	Self-exclusion	• Individual excludes themselves from a range of venue types (e.g., hotels, clubs and casinos that operate electronic gaming machines)	• Self-excluders abstained from most problematic gambling and fewer had • harmful consequences vs. non-excluders • Self-exclusion may have similar short-term outcomes as counselling alone and may reduce short-term harms
Hodgins (2005)^a^ [54]	Workbook	• CBT-based workbook includes self-assessment, goal setting, strategies (e.g., self-exclusion, alternative activity scheduling, cognitive restructuring), maintenance and local treatment resources	• Intervention reduced PG behaviour and reduction was maintained at 12- and 24-months
Hodgins et al. (2009) [55]	Workbook	• CBT workbook includes self-assessment, goal setting, strategies (e.g., self-exclusion, lternative activity scheduling, cognitive restructuring), maintenance and local treatment resources	• Workbook only group participants were just as likely to have significantly reduced their losses over the year and to not meet criteria for pathological gambling as those in brief treatment and brief booster treatment
Hodgins et al. (2001) [56]	Workbook	• CBT workbook includes self-assessment, goal setting, strategies (e.g., self-exclusion, alternative activity scheduling, cognitive restructuring), maintenance and local treatment resources	• Participants who received a motivational enhancement telephone intervention and a self-help workbook (vs. workbook only) had better outcomes than participants in a wait-list control at 3 and 6 month follow-up, but at the 12-month follow-up, the advantage of the motivational interview and workbook condition was found only for participants with less severe gambling problems. Overall, these results support the effectiveness of a brief telephone and mail-based treatment for problem gambling.
Hodgins et al. (2007) [57]	Informational Booklets	• Booklets included topics on dealing with urges to gamble; negative emotions as a cause of relapse; “getting back on the wagon” after a relapse; lifestyle balance; financial issues; stages of change; and dealing with comorbid emotional and addiction problems.	• Participants who received booklet summarizing relapse prevention information had improved PG scores, reduced number of gambling days and dollars lost
Hodgins et al. (2004) [58]	Workbook	• CBT workbook contains five sections: (a) self-assessment, (b) goal setting, (c) strategies, (d) maintenance, and (e) other treatment resources. • Individual completes brief exercises in each section of the workbook.	• Participants who received a motivational telephone intervention plus a self-help workbook had better outcomes (i.e., gambled fewer days, lost less money, and had lower South Oaks Gambling Screen scores) than participants who received only the workbook
Jauregui et al. (2017) [59]	Coping Strategies and Styles • Problem Solving • Cognitive Restructuring • Social Support • Emotional Expression • Problem Avoidance • Wishful Thinking • Social Withdrawal • Self-Criticism	Problem Solving Strategies: • Individual focuses on eliminating stress by modifying the situation that causes it.	• Pathological gamblers (vs non) obtained significantly higher scores in pathological gambling, anxiety, depression, self-criticism, emotional expression, wishful thinking, problem avoidance, social withdrawal, problem disengagement, emotional disengagement, and disengagement
		Cognitive Restructuring: • Individual modifies the meaning of the stressful situation.	
		Social Support: • Individual seeks emotional support from social circle. Emotional Expression: • Individual aims to express the emotions that arise in the stress process.	
		Problem Avoidance: • Individual denies and avoids thoughts and acts related to the stressful situation.	
		Wishful Thinking: • Individual dreams and thinks about a non-stressful reality.	
		Social Withdrawal: • Individual distances themselves from significant people associated with the emotional reaction of the stressful situation.	
		Self-criticism: • Individual engages in self-blame and self-criticism concerning the stressful situation itself and how one dealt with it.	
Labrie et al. (2012) [60]	Self-help toolkit	The toolkit provides: • Exercises such as cost-benefit analysis of gambling. • Information about managing urges, managing change that comes with dealing with an addiction, what makes gambling problematic, and gambling facts. • Specific directives about how to quit (i.e., skills building). • Encouragement for people to practice quitting while simultaneously preparing them for failed attempts (i.e., relapse prevention).	• Toolkit recipients reported recently abstaining from gambling • A self-help toolkit and other self-directed resources can assist in remediating gambling-related problems for individuals who do not want formal treatment
Ladouceur et al. (2000) [61]	Self-exclusion	• Individual excludes themselves from a government operated Canadian casino for periods that range from 6 months to 5 years.	• 30% of participants (95% of participants were severe pathological gamblers) reported that they completely stopped gambling once enrolled in the self-exclusion program
Ladouceur et al. (2007) [62]	Self-exclusion	• Three self-exclusion programs in Quebec Casinos (Montreal, Gatineau, and Charlevoix), in which individuals exclude themselves from casinos for periods ranging from 6 months to 2 years.	• The self-exclusion program facilitated positive results including: reduced urges to gamble, reduced DSM score, reduced intensity of negative consequences for gambling (daily activities, social life, work, mood) and an increased perception of control • At the 6-month follow-up, more than half the participants had breached their contract or returned to a casino • Self-exclusion detection system needs to be improved
Lalande and Ladouceur (2011) [63]	Limit-setting	• Person sets a strict money limit before beginning a gambling session and an intention to quit once they reach the limit.	• Both the PG and non-PG groups use monetary loss limit as a form of self-control to avoid overspending • Those in the PG group set a higher limit than those experiencing NPG; those experiencing PG continue spending after reaching their limit, while those who were not engaged in PG stop gambling when reaching their limit • Internal, external, implicit and explicit limits are proposed to operationalize self-control and self-regulation during gambling sessions
Linardatou et al. (2014) [64]	Stress management • Relaxation Breathing • Progressive Muscle Relaxation	Relaxation Breathing: • Individual engages in a controlled method of breathing to induce relaxation.	• The intervention group demonstrated statistically significant improvements in stress, depression, anxiety symptoms, life satisfaction and better daily routines • Stress management may provide psychosocial benefits and improve the well-being of individuals with pathological gambling • It can be incorporated into PG programs
		Progressive Muscle Relaxation: • Individual engages in consecutive contractions and relaxations of different muscle groups (skeletal, facial and respiratory) in a down-top orientation to induce relaxation. • A CD guides them through the relaxation techniques (10 min RB, 15 min PMR); the CD instructs them to perform the techniques twice a day for 8 weeks at home (maximum 112 sessions) and to notice the difference between being tense and relaxed to improve perception of the relaxation response.	
Luquiens et al. (2016) [65]	Self-help book (Workbook)	• The CBT workbook includes content on motivation, financial issues, cognitive distortions, triggers, life reorganization and relapse prevention.	• No significant difference in efficacy between the group with guidance compared to the group without guidance and control group • Internet-based CBT should include intrinsic motivational components to increase engagement
Martin (2013) [66]	Self-help online toolkit	• Toolkit is designed to help individuals figure out if they need to change their gambling behaviour and decide how to deal with the process of change. • It contains exercises to help determine the costs and benefits of gambling and information on managing urges.	• When completed by a large number of people, online health surveys may be advantageous for screening, intervening and providing self-help information for disordered gambling
Moore et al. (2012) [67]	Various • Cognitive Approaches • Direct Action • Social Experience • Avoidance • Limit Setting	Cognitive Approaches: • Individual re-orders their priorities with respect to gambling, thinks about it differently and focuses on different things.	• Problem gamblers who were trying to reduce their gambling were more likely to use strategies such as Cognitive Approaches, Direct Action, Social Experience, Avoidance and Limit Setting than other gambler groups
		Direct Action: • Individual uses strategies such as cutting up credit cards or self-exclusion.	
		Social Experience: • Individual maximizes the likelihood that spending time at gambling venues would be socially oriented rather than gambling focused.	
		Avoidance: • Individual avoids gambling venues and places personal restrictions on their access o money at venues.	
		Limit Setting: • Individual sets a strict money or time limit before beginning a gambling session and quits gambling once they reach that limit.	
Nelson et al. (2010) [68]	Self-exclusion	• Individual enrolled in the Missouri self-exclusion program are responsible for not entering any casinos in the state.	• Enrolment in the self-exclusion program reduced gambling behaviour at 6 months
Subramaniam et al. (2017) [69]	Responsible gambling strategies • Delayed Gratification • Setting Limits • Maintaining Balance	Delayed gratification: • Individual quells the need for immediate results.	• The main theme of responsible gambling was comprised of two themes: self-development strategies and limit gambling related harm and family interventions to reduce the harm from gambling • Subthemes included delayed gratification, perception of futility of gambling, settling limits, maintaining balance, help-seeking and awareness of disorders gambling in self or others • Families play a significant role in Asian societies in imposing RG, education and counseling of families is important
		Setting limits: • Individual sets time or ‘money’ limits on themselves.	
		Maintaining balance: • Individual maintains a sense of balance in terms of their behaviour; curbs their excessive spending, or time spent gambling.	
Toneatto et al. (2014) [70]	Workbook; Mindfulness	• Workbook contains CBT and CD-guided mindfulness (15-min mindfulness instruction & 30-min practice session). • Mindfulness content contains exercises and information about awareness of breathing; shifting thoughts of gambling; being present-focused; awareness of cognitive processes (especially thoughts about gambling). • CBT content contains instruction in traditional content-focused techniques, such as behavioural problem-solving & cognitive restructuring.	• Compared to a wait list control, the mindfulness intervention significantly reduced the severity of gambling, gambling urges and psychiatric symptoms at end-of treatment • There was a significant decrease in the proportion of the sample meeting criteria for pathological gambling
Townshend (2007) [71]	Self-exclusion	Self-Exclusion in New Zealand • Individual self-excludes themselves from a gambling venue for a period up to 2 years and there may be a fine of$500 if they breach the self-exclusion ban.	• Self-exclusion is an effective treatment tool for participants who have an “extreme difficulty” controlling their gambling using other methods • Self-exclusion may be more effective in a jurisdiction with a public health environment than has been reported in other jurisdictions.
		Self-Exclusion in the U.S. • Regulations on self-exclusion vary by state.	
		Self-exclusion in Canada (British Columbia): • The British Columbia Lotteries Corporation allows for individuals to self-exclude for 6 months, 1, 2 or 3 years and there may be a fine of $5000 if the self-exclusion is violated • This self-exclusion applies to all venues with slot machines, commercial bingo halls across British Columbia and/or the PlayNow locations.	

^a^The same workbook was used in Hodgins (2005), Hodgins et al. (2009), Hodgins et al. (2001)

#### Behavioural strategies

Behavioural self-management strategies are those in which people modify an aspect of their behaviour in order to manage their gambling. Strategies included in this category were self-exclusion [41, 49, 50, 52, 53, 61, 62, 68, 71], money and time limiting [51, 52, 63, 67, 69], alternative activity scheduling [41], direct action [67], social experience [67], delayed gratification [69] and maintenance of balance [69]. While definitions of self-exclusion varied across studies, it was generally defined as entering into formal agreement with a land-based or online gambling venue to be excluded from the venue. In most cases, the terms of the agreement included consequences (e.g., fines, trespassing charges) or restrictions on the collection of winnings (i.e., not allowed to collect winnings) when the agreement was breached. Seven studies examined the effectiveness of self-exclusion for PG and generally reported it to have positive results on its own and in combination with counseling; however, one study reported that over 50% of participants breached self-exclusion agreements within 6 months [62]. Hayer and Meyer [49] reported on the characteristics of people who self-excluded, noting that financial difficulty was the most cited reason for self-exclusion, and that male and middle-aged individuals were most likely to self-exclude. Hing et al. [50] found that people experiencing PG who were involved in problematic internet gambling were less likely to self-exclude from land-based venues (one-fifth of their gambling behavior) and more likely to self-exclude from online gambling sites than their than their land-based counterparts. One study reported that self-exclusion may be more effective in jurisdictions that frame PG as a public health issue because doing so places responsibility on gambling venues instead of people experiencing PG to enforce the ban [71].

Self-limiting strategies with duration of time and the amount of money were described in five studies. Hing et al. [52] described a process in which an individual deposited monetary amounts to bet at the outset of a gambling episode and stopped gambling once that limit was reached. Two studies reported on the effectiveness of limiting strategies, one noting that these strategies predict non-harmful gambling [53] and another reporting limited success [52]. Some evidence indicates that limiting strategies may not be well suited for severe cases of PG. Lalande and Ladouceur [63] reported that those experiencing pathological gambling and those who did not engage in pathological gambling both use money limiting strategies to avoid overspending; however, people experiencing pathological gambling set higher limits and broke these limits more than those who were not experiencing pathological gambling.

Delayed gratification (i.e., quelling the need for immediate results of a gamble) and maintaining balance (i.e., avoiding excesses in behaviour) were self-management strategies reported by older adults (60+) with gambling problems or probable pathological gambling [69]. Alternative activity scheduling (i.e., scheduling non-gambling activities) was effective in reducing PG scores for some women who combined this activity with self-exclusion [41].

#### Cognitive strategies

Cognitive self-management strategies address thoughts, beliefs and cognitions surrounding gambling. Two studies described cognitive restructuring which involves changing irrational or negative thoughts and beliefs about gambling and replacing them with realistic and positive thoughts and beliefs to limit PG. Jauregui at al [59] reported no significant mediating effect of cognitive restructuring on anxiety between those experiencing pathological gambling and those who did not gamble. Moore et al. [67] examined use of cognitive restructuring for self-regulation of gambling among those with (PG) and without (NPG) gambling problems. They were interested in whether gambling status (PG and NPG) and frequency of gambling (low versus high) was associated with use of cognitive restructuring. They found that the PG-high frequency group was most likely to use cognitive restructuring, followed by PG-low frequency, NPG-high frequency, and NPG-low frequency.

#### Coping strategies

Four studies described self-management strategies in the form of coping skills and/or self-directed activities to improve coping skills. Both adaptive and maladaptive strategies were described including mindfulness) [70], emotional expression [59], relaxation breathing [64], progressive muscle relaxation [64], social support [59], problem solving [59], avoidance [59, 67], wishful thinking [59], social withdrawal [59], self-criticism [59], and imaginal desensitization^1^ [48]. Mindfulness and imaginal desensitization reduced gambling severity and gambling urges among a population of people experiencing PG [48, 70]. Maladaptive coping strategies such as avoidance, wishful thinking, social withdrawal, self-criticism, and emotional expression were associated with higher PG scores [59]. In one study, relaxation breathing and progressive muscle relaxation were effective strategies in reducing stress, depression and anxiety, and improving life satisfaction and daily routines (e.g., breakfast and dinner) among people experiencing PG [64].

#### Multi-part interventions

Multi-part self-management interventions provide a variety of tools to help people who want to change their gambling behavior to monitor their gambling activities, set and monitor goals, use self-reflection to recognize underlying motivations and repercussion of their addiction. These interventions include the use of workbooks, self-directed CBT interventions, self-help toolkits, booklets, and personalized feedback tools.

##### Self-directed CBT

Two studies described online CBT interventions for use without the assistance of a therapist [44, 45] such as challenging and replacing erroneous thoughts. They also contained other self-management strategies such as debt management, managing high risk situations, recognizing triggers [44, 45], imaginal desensitization, relaxation training, goal setting, emotions maintenance, relapse prevention [44], psychoeducation, and identifying social consequences of gambling [45]. Casey et al. [44] found that a CBT intervention was associated with reduced gambling severity, other PG and mental health outcomes, and greater life satisfaction after the initial treatment and at 12-months follow-up. The CBT intervention in a study by Castrén et al. [45] was associated with reduced gambling-related problems, urges, impaired control of gambling, social consequences, gambling-related cognitive erroneous thoughts and depression.

##### Workbooks

Nine studies examined online and offline workbooks with exercises meant to manage PG-related outcomes. Although the structure of the workbooks and the topics varied, common elements included motivation to change and the change process [42, 43, 72] and self-reflection and improved self-awareness of gambling related cognitions [43, 70]. Most workbooks contained some CBT content, such as information on cognitive distortions and cognitive- restructuring [43, 54–56, 58, 70]. Others included materials and exercises on goal-setting [42] and finances [65]. Most workbooks provided descriptions and information for other self-management strategies noted in this review such as mindfulness [42, 70], limiting strategies [43], self-exclusion [54–56], stress management [42] and alternative activity scheduling [54–56]. The majority of workbooks included information relating to managing urges and/or relapses, maintenance and resources [42, 43, 54–56, 58, 65]. Some studies suggested that workbook-only interventions were effective in reducing harms associated with PG [42, 54] while other studies noted improved outcomes when the workbook was paired with therapist guidance or other formal support [43, 55, 58]. Generally, the workbook interventions were reported to be well-received by clients and described as an approach to expand PG services to individuals.

##### Booklets and toolkits

Informational booklet [57] and self-help toolkit [60, 66] interventions are similar in structure and content to workbook interventions and were utilized in three studies. Like workbooks, both toolkits and the informational booklets contained resources on managing urges, the change process and relapse prevention. The primary focus of the toolkits was to help individuals self-reflect on their gambling behaviours and included exercises to determine the costs and benefits of gambling behaviour to motivate change. The booklets included additional information on lifestyle balance, financial issues and managing comorbid conditions (e.g., emotional and addiction problems). LaBrie et al. [60] reported improved PG-related outcomes for toolkit recipients and advised that toolkits may be a viable treatment alternative for individuals who do not want to engage in formal treatment courses. Hodgins et al. [57] reported that participants who received repeated mailing of bibliotherapy (relapse prevention booklets) for PG were more likely to meet their gambling-related treatment goals than those who did not receive mailings. However, participants who received repeated mailings did not differ from participants who received a single mailing on gambling frequency or reported gambling losses.

##### Personalized feedback tool

Online personalized feedback tools were noted in two studies [46, 47]. Personalized feedback tools involve some form of self-assessment of PG behaviour and/or ongoing information gathering to provide a personalized profile of PG behaviour, beliefs and habits. This information is then presented back to the user along with a comparison of their behaviour with the gambling behaviour or cognitions of other users and/or general population to establish a risk level for PG. This feedback is presented along with helpful strategies and techniques to lower risk and limit gambling. Additionally, in cases of continual information gathering/behaviour tracking, individuals could opt-in to have personalized messages sent to them when they were engaging in risky gambling behaviours. Cunningham et al. [46] reported a reduction in the number of days gambled for participants receiving a partial feedback tool (i.e., feedback about behaviour without comparison to norm for that behaviour) in comparison to those who received no intervention. They found no evidence to support the efficacy of a normative feedback tool. Forsström et al. [47] found low continued usage of a personalized feedback tool among participants despite positive opinions of its content.

## Discussion

The purpose of this paper was to examine the scope of current published literature on PG self-management strategies. We identified 31 studies and reviewed 23 different self-management strategies for PG, published between 2000 and 2017. In a previous review of self-help for PG (up to April 2008) only two types of self-help had been reported; these were self-help manuals and audiotapes [35]. Our findings show that there is a growing body of literature examining a diverse range of self-management strategies for PG. The most commonly cited strategies in this review were self-exclusion (*n* = 9), workbooks (*n* = 8), and money or time limiting strategies (*n* = 4). Other strategies included various cognitive and behavioural strategies, coping strategies, stress management, and mindfulness. Surprisingly, technological modes of treatment (e.g., virtual reality treatments) were not well-represented in this review. Three studies examining the use of CBT in virtual reality with therapist assistance (therapist-assisted studies were excluded from our review), showed that the technology has promise for the treatment of PG [73]. Although the self-management strategies noted in this review are conceptually similar to those identified in other literature [35–37], the labelling and categorization of strategies was found to considerably vary across studies. Future research examining self-management strategies for PG would benefit from standardized conceptualizations of strategies and shared terminology.

Self-exclusion was the most examined approach to self-management in this review, yet there is little evidence for its effectiveness. In fact, compliance rates are quite low (13 to 30%), with inadequate surveillance and enforcement of bans, and complicated enrollment processes which impede use of this option to manage PG [51, 74]. A deeper understanding of self-exclusion, in particular, and other self-management strategies requires consideration of comorbid health and social concerns [6, 75]. People who experience complex health and social concerns such as homelessness, mental health issues, substance use disorders, and incarceration are at greater risk of PG, yet current services do not address multimorbidity [3, 4]. Notably, although some studies in this review included participants with mental health and/or substance use comorbidities, most did not explicitly address these comorbidities with PG (e.g., alcoholism) in the design of self-management strategies. Further research is needed to explore the complex interplay between PG and comorbid conditions and design comprehensive interventions that address multiple needs [6, 75].

In addition, a key finding from this review was a lack of research examining self-management approaches tailored to specific socio-demographic sub-groups (e.g., age, income, gender, ethnicity, geography) [49]. There were few studies among younger populations aged 18 to 35 years old. Only one study focused on older adults who were aged 60 and above [69]. Older adults have their own unique concerns that may affect how they use self-management strategies and the types of strategies that they prefer (e.g., access to and familiarity with technology) [76]. Only one study examined participants with low-income backgrounds [43]. While many studies had mixed-sex samples only one study considered a gendered approach to self-management strategies. One study reported that, within Asian culture, families use a variety of mechanisms to enforce responsible gambling (e.g., family exclusion orders) [69]. Research is needed to understand what self-management approaches may be appropriate and effective for a variety of populations.

Many empirically validated theories of behaviour change, including diffusion of innovations [77], social cognitive theory [78], and the social ecological model [79] assert that social relationships play a significant role in facilitating behavioural change. These findings suggest that we need a better understanding of the role that support networks/circles, and peer support outside the formal treatment environment may play in PG self-management [76, 80].This topic deserves specific exploration of those strategies that may or may not be effective for specific populations, such as people facing poverty and homelessness and those from varying ethnic cultures. This could be accomplished using a realist perspective to understand what works, for whom, and under what conditions [81, 82]. Given the majority of people experiencing PG do not actively seek treatment [13, 14], offering evidence of the effectiveness of personal approaches to self-management is imperative. Moreover, past research suggests that one-third to upwards of 82% of people experience natural recovery from PG with men more likely to report this happening than women [19, 83, 84]. While the literature on PG self-management strategies has evolved since Raylu et al’s paper [35], there is still little evidence of the effectiveness of self-management to reduce harms associated with gambling.

### Strengths and limitations

This study has a few limitations that should be noted. It is also possible that some relevant articles were missed, as only articles published between January 1, 2000 and June 28, 2017 were included as well as those published in English. We included French publications in our initial search with the idea that we would review English translations of papers written in French, but no translations were available. None of the authors are proficient in French language. However, our search strategy was comprehensive and guided by an information science specialist. To our knowledge, this is the first review of self-management strategies for PG since a previous review in 2008. The earlier review focused on broad definition of self-help that included forming partnerships with heath care providers [25]. We defined self-management with a narrower scope focusing on self-care outside the health and social service system as we were specifically interested in strategies that people manage on their own (or after active treatment) to reduce personal harms associated with gambling. Our review focused on adults; however, PG among teens and young adults is a serious public health concern. As such it would be prudent to similarly explore self-management among those under age 18 [85, 86]. This is especially important given that gambling seems to differ on a variety of dimensions among youth compared to adults, including reasons for gambling, comorbidities, and consequences of gambling [85, 87–90]. Individual capacity is an important consideration in any approach to care for gambling concerns, whether through professional treatment or self-care. This becomes complicated by cognitive and intellectual disabilities, multimorbidities, and such social determinants of health as homelessness and poverty. In particular self-management may not be an appropriate approach to care.

## Conclusions

Given that it is the minority of people with gambling concerns that seek treatment, that stigma is an enormous barrier to care, that PG services are scarce and do not address multimorbidity [5, 6, 14, 91, 92], it is imperative that we examine the personal management of gambling as an option to formalized treatment. This is the first review to examine self-management of PG and findings indicate that evidence is lacking on this topic. It is imperative that the field explore self-management in PG in more depth and for specialized populations to understand the nuances of recovery for diverse populations.

## Additional files

Additional file 1: PRISMA-ScR Checklist (DOCX 271 kb)

Additional file 2: Medline Search Strategy (DOCX 15 kb)

## Abbreviations

CBT: Cognitive Behavioural Therapy
EGM: CD: Compact Disk
IDMI: Imaginal Desensitization plus Motivational Interviewing
M: Mean
N: Number
NPG: Non-problem gambling
PG: Problem Gambling
PGSI: qProblem Gambling Severity Index
SD: Standard Deviation

## Footnotes

1 Imaginal desensitization is an approach through which patients learn to visualize a situation that triggers an impulse to carry out an impulsive behavior (e.g., gambling), and then imagine overcoming the urge using learned relaxation skills. The idea is that with time, an individual gains confidence in their ability to manage urges and becomes “desensitized” to triggering situations.
