# Supplementary figures and images for: The use of self-management strategies for problem gambling: a scoping review
Source: BMC Public Health. 2019 Apr 29;19:445. doi: 10.1186/s12889-019-6755-8 (PMC6489359; doi:10.1186/s12889-019-6755-8)

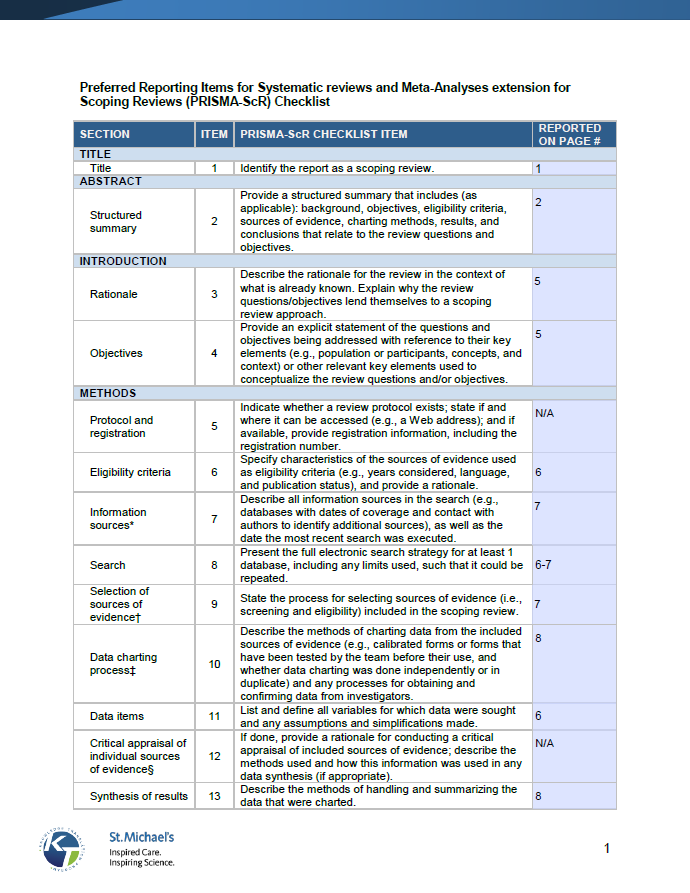


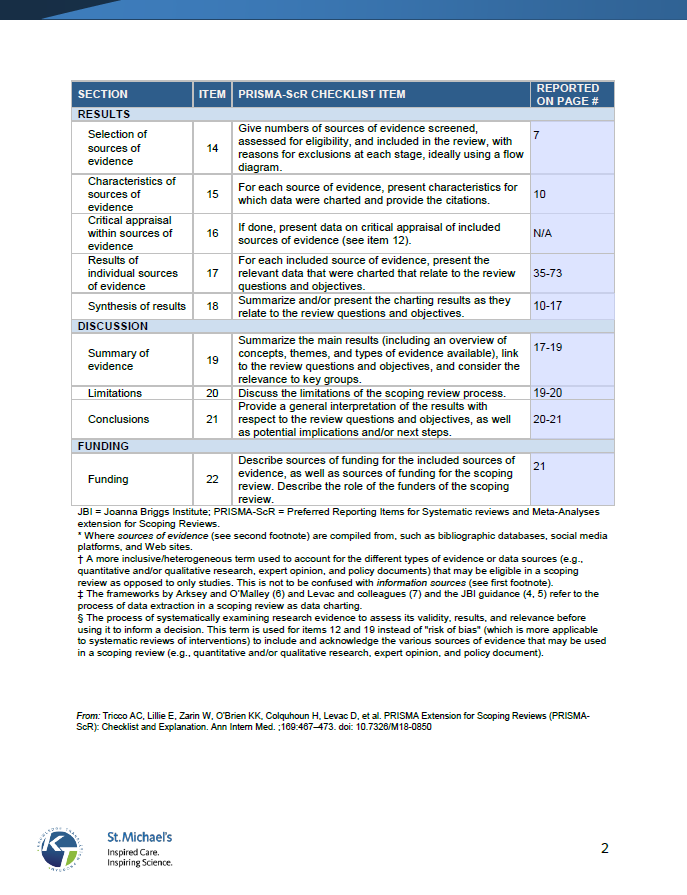

Supplement: Supplementary file 1 — PRISMA-ScR Checklist (DOCX 271 kb) [file 12889_2019_6755_MOESM1_ESM.docx]
