# Supplementary material for: The use of self-management strategies for problem gambling: a scoping review
Source: BMC Public Health. 2019 Apr 29;19:445. doi: 10.1186/s12889-019-6755-8 (PMC6489359; doi:10.1186/s12889-019-6755-8)
Supplement: Supplementary file 2 — Medline Search Strategy (DOCX 15 kb) [file 12889_2019_6755_MOESM2_ESM.docx]

**Supplementary File: Medline Search Strategy**

**Database: Ovid MEDLINE: Epub Ahead of Print, In-Process & Other Non-Indexed Citations, Ovid MEDLINE® Daily and Ovid MEDLINE® <1946-Present>**

Search Strategy:

--------------------------------------------------------------------------------

1 Gambling/

2 (gamble* or gambling*).tw,kf.

3 casino*.tw,kf.

4 slot machine*.tw,kf.

5 betting.ti.

6 (lottery or lotteries or sports bet* or wagering or online bet* or dog bet* or horse bet* or race bet* or off-course bet* or sports bet* or horse racing or bingo or poker or betting machine* or pokies or scratch ticket* or scratch card* or track bet* or electronic gaming machine* or video lottery terminal* or sports lotter* or table games or betting on the horses or horse race bet* or off track bet* or craps or roulette or blackjack).tw,kf.

7 1 or 2 or 3 or 4 or 5 or 6

8 Self Care/

9 self efficacy/

10 exp Adaptation, Psychological/

11 Self-Control/

12 Resilience, Psychological/

13 self help.tw,kf.

14 self-monitor*.tw,kf.

15 self-manage*.tw,kf.

16 self regulat*.tw,kf.

17 self control.tw,kf.

18 self-guided.tw,kf.

19 self-taught.tw,kf.

20 self educat*.tw,kf.

21 self efficacy.tw,kf.

22 self-recovery.tw,kf.

23 self-therapy.tw,kf.

24 self treatment.tw,kf.

25 self care.tw,kf.

26 (self exclusion or self exclud*).tw,kf.

27 empower*.tw,kf.

28 (resilience or resiliency).tw,kf.

29 natural recovery.tw,kf.

30 (limit setting or set limits).tw,kf.

31 money limit* strateg*.tw,kf.

32 coping strateg*.tw,kf.

33 exp peer group/

34 Self-Help Groups/

35 (gambl* anon* or gam anon).tw,kf.

36 (mutual aid or mutual support or peer support or 12-Step group* or 12-Step program* or Twelve-step program* or Twelve-step group* or support group* or 12 steps or twelve steps or group support* or recovery group* or nonprofessional support or non-professional support or peer to peer or peer counsel*).tw,kf.

37 controlled gambling.tw,kf.

38 responsible gambling.tw,kf.

39 online support.tw,kf.

40 online community.tw,kf.

41 community forum*.tw,kf.

42 online forum*.tw,kf.

43 online group*.tw,kf.

44 exp social support/

45 Harm Reduction/

46 harm reduction.tw,kf.

47 Meditation/

48 Mindfulness/

49 Computer-Assisted Instruction/

50 Mobile Applications/

51 (thought record* or journaling or diary or diaries or worksheet* or cognitive strateg* or urge surf* or strengths based or mindfulness).tw,kf.

52 (smartphone app* or smart phone app* or mobile phone app* or mobile app* or mhealth or e-mental health or online app* or internet app* or e-health or web app* or ipad app* or iphone app* or internet based or internet delivered).tw,kf.

53 or/8-52

54 7 and 53

55 limit 54 to (english or french)

56 55 not (animals/ not humans/)

57 limit 56 to (case reports or comment or editorial or letter)

58 56 not 57

59 limit 58 to yr="2000 -Current" [June 28 2017]
